# Cellulose-Based Films with Ultraviolet Shielding Performance Prepared Directly from Waste Corrugated Pulp

**DOI:** 10.3390/polym13193359

**Published:** 2021-09-30

**Authors:** Guangmei Xia, Qiwen Zhou, Zhen Xu, Jinming Zhang, Xingxiang Ji, Jun Zhang, Haq Nawaz, Jie Wang, Jianfeng Peng

**Affiliations:** 1 State Key Laboratory of Biobased Material and Green Papermaking, Qilu University of Technology, Shandong Academy of Sciences, Jinan 250353, China; biliqwzhou@163.com (Q.Z.); xz521953@qlu.edu.cn (Z.X.); WJ05111129@163.com (J.W.); pjf1966614429@163.com (J.P.); 2 Beijing National Laboratory for Molecular Sciences, CAS Key Laboratory of Engineering Plastics, Institute of Chemistry, Chinese Academy of Sciences (CAS), Beijing 100190, China; jzhang@iccas.ac.cn (J.Z.); haqnawaz@bjfu.edu.cn (H.N.)

**Keywords:** corrugated cartons, recycle, cellulose-based films

## Abstract

As the most important paper packaging materials, corrugated cartons with a tremendous amount of production demonstrate several advantages and have been widely used in daily life. However, waste corrugated cartons (WCCs) are usually recycled and reused to produce new corrugated cartons, and their properties are decreased dramatically after several cycles. Therefore, recycling and converting WCCs into cellulose-based film with high value is attractive and significant. Herein, without any pretreatment, the waste old corrugated cartons were directly dissolved in ionic liquid 1-allyl-3-methylimidazolium chloride, and semitransparent cellulose-based films were successfully fabricated. It was indicated that cellulose-based films displayed better UV-shielding property and hydrophobicity than traditional cellulose films. Interestingly, the cellulose-based films regenerated from deionized water displayed higher tensile strength, elongation at break, and toughness. Their tensile strength could reach 23.16 MPa, exhibiting enormous superiority as wrapping and packaging materials to replace the petrochemical polyethylene membrane (8.95 MPa). Consequently, these renewable, biodegradable, and high-valued cellulose-based films were successfully fabricated to simultaneously realize the valorization of old corrugated cartons and supplement the petrochemical plastics.

## 1. Introduction

Petrochemical plastics, which are cheap, convenient, and durable materials, have been widely used in various fields, such as toys, electronics, furniture, construction, packaging, and wrapping [1,2,3,4]. However, most petrochemical plastics are incinerated or landfilled at the end of their service life. It has been reported that 12% of approximately 6.3 billion tons of plastic waste was incinerated, while the plastic waste accumulated in nature or landfills was about 79% by 2015 [1]. Unfortunately, some of the plastic wastes have been transported from inland to oceans and then amassed quickly in our food chain. Moreover, they are difficult to decade due to their stable polymer chains, leading to pollutions to our environment [2,5,6]. Therefore, developing low-cost, biodegradable, and environmentally friendly materials to supplement or even replace petrochemical plastics is an urgent and meaningful task.

Renewable and biodegradable biomass resources have attracted considerable attention in recent years [4,7,8,9,10] and will develop rapidly with the goal of carbon neutrality set worldwide to address the global catastrophic climate crisis [11,12,13]. Generally, plants biomass resource is a good choice for the production of renewable, biodegradable, and environmentally friendly materials [14,15,16,17]. As one of the three major components in plants biomass resources, cellulose is ubiquitous in nature and has been widely converted into various cellulose derivatives and cellulose materials [7,8,18], showing great potential in fluorescent smart materials [19], food packaging [18], 3D printing technology [8], triboelectric nanogenerators [20], etc. It is worth noting that regenerated cellulose materials are attractive candidates to replace traditional petrochemical plastics [4]. Nevertheless, the cotton linter (12–15 %) and wood pulps (85–88%) are still the major components of cellulose sources in the industry [21], significantly limiting the application and development of cellulose materials, not to mention replacing the nonbiodegradable petrochemical plastics. In contrast, abundant and low-cost cellulose sources, such as agricultural, forestry, industrial, and domestic wastes, can be promising raw materials for cellulose. Meanwhile, most of the regenerated cellulose materials display poor ultraviolet shielding performance and absorb moisture easily, decreasing their service life. Therefore, developing cellulose products with functional properties to meet the increasing social demands is urgent and significant [15,22].

Copious studies have been initiated to fabricate cellulose products from the wastes to meet circular economy and sustainable development [21]. Cellulose nanofibers (CNFs) have attracted extensive studies, due to their unique structure and excellent property [23]. Several previous works have demonstrated that CNFs can be produced from waste paper materials such as recycled newspapers, mixed office paper, and waste paper separated from mixed municipal solid waste, which enhanced the usage of secondary raw materials and converted low-quality waste fibers into value-added materials [23,24,25]. It is reported that deep eutectic solvent (DES) has the ability to dissolve lignins and can disrupt the hydrogen bonding between cellulose fibers [4]. By using DES as a solvent through in situ lignin regeneration method, Hu et al. fabricated a strong, biodegradable, and recyclable lingocellulosic bioplastics from agri-biomass recently, which showed high mechanical strength, good ultraviolet-light resistance, water stability, and thermal stability [2,4]. It is worth noting that transparent cellulose-based materials with good mechanical properties can be obtained by dissolution and regeneration processes. It is well known that cellulose is recalcitrant biomass and is difficult to dissolve in most organic solvents. The traditional viscose process is still the most popular method in the industry, and some solvent systems have also been found for producing regenerated cellulose materials, such as LiCl/N,N-dimethylacetamide system [26], NaOH/thiourea, or NaOH/urea [27], N-methylmorpholine-N-oxide [28], ionic liquids system [29]. As a new type of cellulose nonderivative solvent, room temperature ionic liquids (ILs) demonstrate many advantages, such as good dissolving capacity, recoverability, etc. Therefore, ionic liquids (ILs) have been widely employed to achieve cellulose-based materials. Several interesting and significant studies on cellulose have been reported by Zhang et al. including the transformation of cellulose-contained wastes such as waste newspapers [18], spent tea leaf [30], agricultural wastes [31,32], and waste cotton textiles [22,33] into cellulose films, which demonstrated good transmittance and mechanical properties, providing a promising alternative to petrochemical packaging materials. However, to the best of our knowledge, few works exhibited cellulose-based products fabricated directly from the waste corrugated pulp.

As the most important paper packaging materials, corrugated cartons with several advantages such as high strength, easy processing, light weight, low cost, convenient transportation and storage, excellent printing adaptability, etc. have huge production and are widely used in daily life. It was reported that 20 million tons of corrugated cartons were consumed domestically per year in the US from 2010 to 2013, while it exceeded 22.5 million tons and arrived at 22.8 million tons in 2020 [34]. However, waste old corrugated cartons are usually recycled and reused to produce new corrugated cartons, and their properties decrease dramatically after several cycles. Hence, other uses instead of papermaking should be more suitable for these waste corrugated fibers. Moreover, few studies have been reported to prepare cellulose-based materials from WCCs [35]. Wang et al. successfully prepared ultra-long cellulose nanofibers through a series of chemical and mechanical treatments, and further fabricated nanopapers after filtration by using the obtained cellulose nanofibrils, which seemed to be strong candidates for fabricating solar cells, panel sensors, and optical electronics [35]. Corrugated cartons are comprised of cellulose fibers, lignin, hemicellulose, and other additives, and lignin gives a brown appearance and possesses many features [34,36]. It was found that lignin was a low-cost source to prepare UV-shielding biomaterials for protection usages, having the advantages of sustainability and biocompatibility due to the abundance of phenolic groups [36,37]. Nevertheless, lignin should be separated from the biomass before usage, which is complex and time consuming, leading to further aggregation, etc. Moreover, most works were focused on exploiting cellulose fibers from WCCs, which caused the lignin resources to be undervalued and underused. Therefore, complete recycling and valorization of waste corrugated cartons within one process is economical and desirable.

In this work, old waste corrugated cartons were dissolved in ionic liquid AmimCl directly and effectively without any pretreatment. Then, the semitransparent cellulose-based films demonstrating good mechanical and ultraviolet shielding performance were obtained after dissolution, coagulation, and regeneration processes. Furthermore, the differences in crystalline property and structure between cellulose-based films regenerated in water and ethanol coagulation baths were also surveyed. Therefore, this work firstly demonstrated the cellulose-based films with ultraviolet shielding performance fabricated directly from waste corrugated pulp via a feasible and eco-friendly process.

## 2. Materials and Methods

### 2.1. Materials

The old waste corrugated milk cartons (WCCs) were collected from households. Cotton pulp (CP) was offered by Shandong ICCAS-Henglian Biobased Materials Co., Ltd., Weifang, China and the degree of polymerization was 530. The 1-allyl-3-methylimidazolium chloride (AmimCl) ionic liquid was produced by the method that has been reported in our work [38]. Deionized water was homemade and all other chemical reagents were bought from Jinan Hengyou New Material Technology Co., Ltd. (Jinan, China) and used without further purification.

### 2.2. Dissolution and Valorization of Old Waste Corrugated Cartons

**Figure 1:**
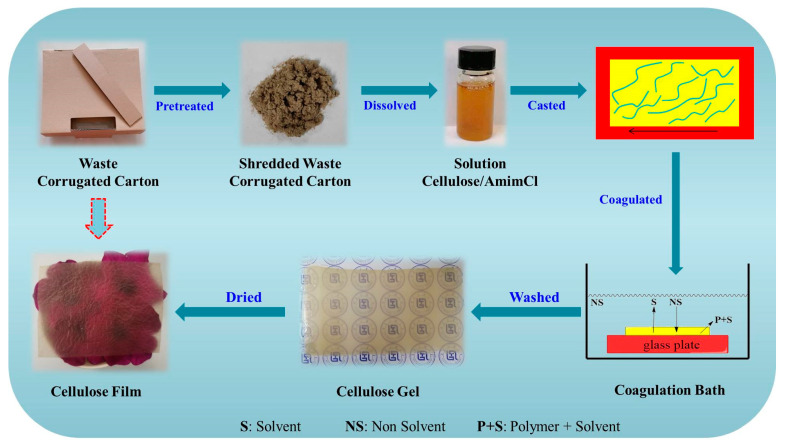
Preparation scheme of cellulose-based films from old waste corrugated cartons.

The process of converting old waste corrugated cartons (WCCs) to cellulose-based films using ionic liquid as a solvent is displayed in Figure 1. Firstly, WCCs were shredded into pieces to enhance the dissolution process. Then, 49 g of AmimCl and 1 g of WCCs were mixed together and heated at 80 °C for 4 h with vigorous mechanical stirring to obtain a homogeneous WCCs/AmimCl solution mixture (2 wt%). Subsequently, the WCCs/AmimCl solution was centrifuged to dispose of the bubbles in the solution and then cast onto a glass plate. The glass plate with a 1 mm thick WCCs/AmimCl solution layer was put into the deionized (DI) water or ethanol coagulation bath to obtain cellulose gels (named alcogel and hydrogel). The cellulose gels were washed many times to dispose of AmimCl in the gels. Finally, the cellulose gels were put into the Kessel paper dryer at 100 °C for 10 min to fabricate the cellulose-based films (Film-E and Film-W). For comparison, the C-gel and C-film were also fabricated by dissolving the CP into AmimCl solvent, in which DI water was used as a coagulation bath.

### 2.3. Characterization

#### 2.3.1. The Degree of Polymerization (DP) of Cellulose in WCCs

The degree of polymerization (DP) of cellulose in WCCs was determined by adopting Ubbelodhe viscometry and cupriethylenediamine as the solvent at 25 °C, which was described in the Standard Test Method for Intrinsic Viscosity of Cellulose (ASTM D795-13), in which the DP of cellulose in WCCs was 297.

#### 2.3.2. Polarized Optical Microscopy of WCCs/AmimCl Solution

The solubility of WCCs in AmimCl was analyzed by PM6000 polarizing microscope (POM) purchased from Nanjing Jiangnan Yongxin Optical Co., Ltd., Nanjing, China. The WCCs/AmimCl solution was put between a clean coverslip and a clean glass slide to probe the dissolution capability.

#### 2.3.3. Ultraviolet and Visible (UV–Vis) Spectra of the Cellulose-Based Films

The UV–Vis spectra of cellulose-based films were recorded by the Ultraviolet Spectrophotometer UV 2600 purchased from Shimadzu, Tokyo, Japan. 

#### 2.3.4. Mechanical Testing of the Cellulose-Based Films

The tensile strength of the cellulose-based films was decided by the TA.XT Plus C texture Analyzer (StableMicroSystem, Surrey, UK), in which the drawing speed was at 4.8 mm min^−1^, and the width and length were 10 mm and 45 mm, respectively. A gauge length was kept 20 mm, and six specimens were measured for each sample. Then, the average value was output. The ASTM D-882 standard was used as the reference for the mechanical test.

#### 2.3.5. Morphology of the Cellulose-Based Films

The morphology of the cellulose-based films was characterized by scanning electron microscopy using an EM-30 Plus microscope (SEM, COXEM, Daejeon, Korea). To obtain the cross-sectional images of cellulose-based films, samples were usually quenched in liquid nitrogen. Before observation, all samples had to be sputter-coated with platinum.

#### 2.3.6. Wide-Angle X-ray Diffraction (WAXD) of the CP, WCCs, and Cellulose-Based Films

X-ray diffraction patterns were recorded by an X-ray diffractometer (D8 ADVANCE, Bruker, Rheinstetten, Germany), in which the scan speed was at 8°/min from 5° to 60° (2θ), and CuKa radiation (λ = 1.5406 Å) were 40 kV and 40 mA.

#### 2.3.7. Wide-Angle X-ray Diffraction (WAXD) of the CP, WCCs, and Cellulose-Based Films

The chemical structure of samples was studied by the attenuated total reflectance Fourier transform infrared spectrometer (ATR-FTIR ALPHA, Bruker, Rheinstetten, Germany), using a resolution of 4 cm^−1^ and obtaining 32 scans. Three spots were detected for each sample, and ethanol was used to clean the Ge crystal between samples. Finally, the OPUS software was employed to analyze the results.

#### 2.3.8. Thermogravimetric Analysis (TGA) of the CP, WCCs, and Cellulose-Based Films

The thermal decomposition behavior of the raw materials and regenerated cellulose films were investigated by the thermogravimetric analyzer (TA Q50, New Castle, DE, USA), which had a precision balance and a ceramic pan inside the furnace. The films were cut into small pieces, and approximately 5 mg of samples was placed in the crucible pot. The samples were heated under a nitrogen atmosphere at a heating rate of 10 °C/min ranging from 50 °C to 800 °C. 

#### 2.3.9. The Surface Hydrophilicity of the Cellulose-Based Films

The OCA 50 (Dataphysics, Filderstadt, Germany) was employed to study the hydrophilicity of the cellulose-based films, and the water contact angles of film W, film E, and C film were recorded. Three to five spots were detected for each sample and the average value was displayed. The time-dependent evaluation of the contact angle of C film, film W, and film E was also recorded.

#### 2.3.10. The Surface Roughness of the Cellulose-Based Films

Ra is the arithmetical mean roughness of the film surface recorded with the Jitai RT200 roughness measuring instrument (Shanghai, China), and three positions were detected for each sample.

## 3. Results and Discussion

### 3.1. Pretreatment and Dissolution of WCCs

**Figure 2:**
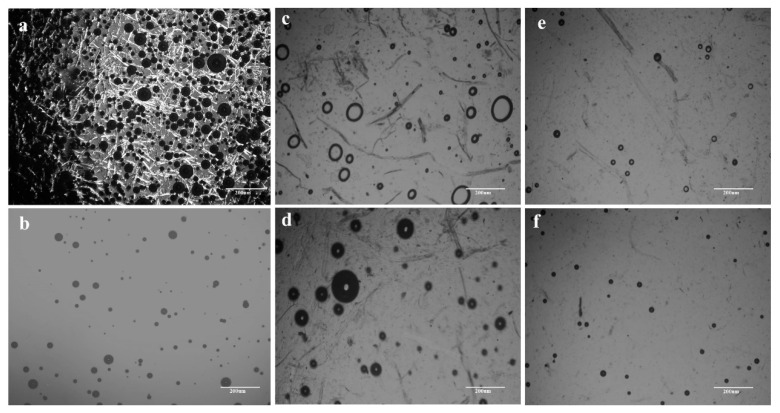
(**a**–**d**) POM micrographs of CP/AmimCl solution at 80 °C after (**a**) 0 min and (**b**) 60 min, and WCCs/AmimCl solution at 80 °C after (**c**) 0 min, (**d**) 30 min, (**e**) 60 min, and (**f**) 240 min.

As a new green nonderivative solvent for cellulose, ionic liquids (ILs) have many advantages, such as easy recyclability, thermal stability, superior dissolving capacity, etc. [29]. Furthermore, it has been proved that AmimCl is one of the most popular ionic liquids to dissolve lignocellulose, and lignocellulose can also be dissolved efficiently in AmimCl [39,40]. The detailed information of the pure cotton pulp and waste corrugated cantons dissolution process in AmimCl was recorded by a polarizing microscope, which is displayed in Figure 2a–f. It is obvious that CP and WCCs contained copious microfibers and the diameters of microfibers were ranging from 10 μm to 50 μm (Figure 2a,c). Meanwhile, most microfibers became short after shredding. Pure cotton fibers can be dissolved completely after 60 min (Figure 2b), while the microfibers of WCCs were swollen and their microfiber profiles became vague (Figure 2d,e) as time passed, indicating that the microfibers of WCCs were dissolved slowly in AmimCl involving swelling and dissolution steps. Eventually, the number of WCCs fibers was decreased obviously and most fibers were disappeared after 4 h stirring at 80 °C (Figure 2f), suggesting that most WCCs fibers were completely soluble in AmimCl. However, the dissolution time of WCCs was longer than that of CP, and some minor parts can also be seen from the POM after 4 h in this work, which can be attributed to the impurities stuck to the WCCs fibers. 

Generally, WCCs contain cellulose, hemicellulose, lignin, and other additives, but cellulose is their major component. Investigations to develop new solvent systems and illuminate the dissolution mechanism of cellulose in different solvent systems have been conducted during the past several decades [41,42]. It was generally accepted that both cations and anions of ILs show a synergistic effect in the dissolution process, promoting the dissolution of cellulose [22,43,44]. It is worth noting that the dissolution mechanisms are complex, and the amphiphilic nature is probably important [42]. Meanwhile, the dissolution mechanism of all the components of lignocellulose in the AmimCl solvent system is complicated, and until now it is not very clear, which needs to be further investigated. Moreover, the viscosity of cellulose/AmimCl solution is very high, leading to numerous bubbles in POM images. Therefore, it is necessary to remove these bubbles before preparing cellulose materials.

### 3.2. Transparency of Cellulose-Based Films

**Figure 3:**
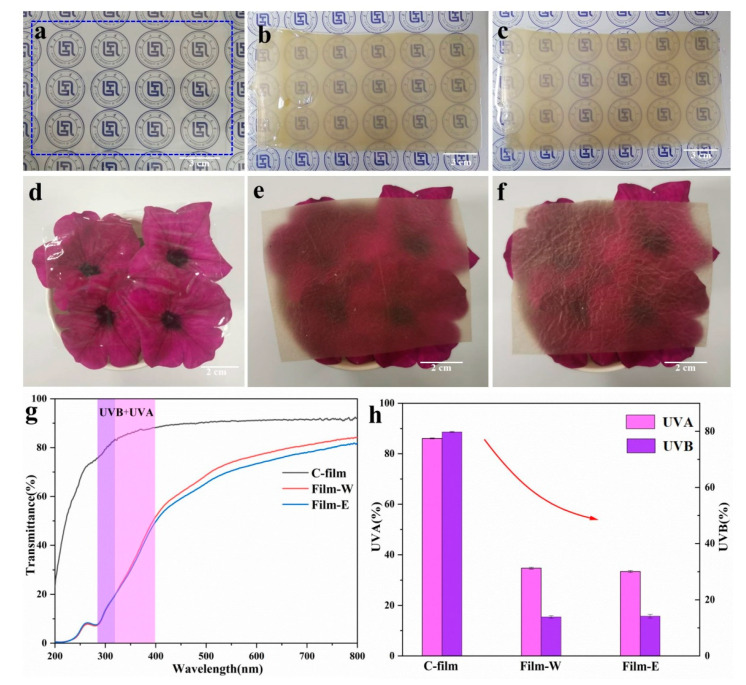
Optical photographs of cellulose-based gels (**a**, C gel; **b**, hydrogel; **c**, alcogel) and cellulose-based films (**d**, C-film; **e**, Film-W; **f**, Film-E); (**g**) UV–Vis spectra, (**h**) UVA and UVB of C-film, Film-W and Film-E.

The application of packaging materials is influenced by their transparency, and Figure 3 demonstrates the optical photographs, UV–Vis curves, UVA, and UVB of regenerated cellulose-based materials. It is can also be seen that transparent cellulose gels and films can be prepared from the pure cotton pulp (Figure 3a,d), and the texture of cellulose-based films fabricated from WCCs is homogeneous, indicating that lignocellulose can be dissolved efficiently in AmimCl. Meanwhile, it is obvious that the cellulose-based gels (Figure 3b,c) and films (Figure 3e,f) are brown and semitransparent. The UV–Vis spectra of Film-E and Film-W in the visible region (400–800 nm) quantifiably describe this performance (Figure 3g). The transmittance of Film-E and Film-W is lower than that of C-film, a product of a high-quality cotton liner, because lignin or other inorganic impurities descending from WCCs are still confined in regenerated cellulose-based gels and films. Moreover, there are minor differences in transmittance between Film-E and Film-W, suggesting that coagulation bath plays a role in the transparency of cellulose-based films, which is also in correspondence with our previous study [22]. However, the cellulose-based films (Film-E and Film-W) show better UV-shielding properties than the traditional cellulose film (C-film), and the UVA and UVB of C-film, Film-W and Film-E are 86.08, 34.68, 33.42 and 79.77, 13.9, 14.14, respectively, because lignin contained copious phenolic groups and is a natural anti-UV radiation substance (Figure 3f) [36]. Moreover, these cellulose-based films can be degraded completely in the environment after use, which is good for humans and the environment [2,4]. In short, although the transmittance of cellulose-based films fabricated from WCCs is lower than that of the traditional high-quality cellulose film, the inexpensive cellulose-based films possess good UV shielding, showing their superiority in renewable, degradable, and ultraviolet shielding packaging and wrapping fields.

### 3.3. Structure and Crystallinity

**Figure 4:**
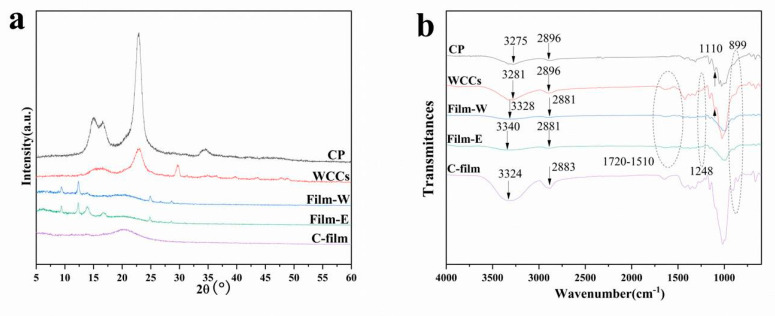
(**a**) XRD and (**b**) FTIR profiles of CP, WCCs, C-film, Film-W and Film-E.

Both X-ray diffraction and FTIR studies were conducted to detect the crystallization properties and structural changes of the regenerated cellulose-based materials and raw materials. Figure 4a presents the X-ray diffraction patterns of WCCs, CP, and cellulose-based films C-film, Film-W and Film-E to achieve detailed information of crystalline phase changes. It can be derived that the high-grade CP shows obvious peeks at around 2θ = 15.1°, 16.8°, 22.8°, and 34.5° corresponding to the crystal planes (1–10), (110), (200), and (004) of cellulose I [18,45]. Therefore, the natural cellulose is cellulose I, and WCCs also display obvious peeks of cellulose I. Generally, the regenerated materials usually demonstrate a wide peak ranging from 15° to 25°, attributed to the overlapped peak of cellulose II two peaks at 21.9° (200) and 20.1° (110), and the amorphous cellulose peak at 17.3°, because of a crystal change of cellulose I to II after the natural cellulose regeneration process [18,22]. C-film displays the obvious cellulose II phase, and cellulose-based films prepared from WCCs (Film-W and Film-E) also show this phenomenon. However, compared with CP, the crystal transformation of cellulose I to II is not very clear for WCCs, and both Film-W and Film-E exhibit some obvious and minor peeks assigned to the impurities. WCCs are usually made from waste recycled paper, and thereby, some impurities still retain in the regenerated-based films. In addition to a crystal transformation after CP and WCCs regeneration, the diffraction peak intensity of cellulose-based films decreases prominently, compared with that of CP and WCCs, indicating the decrease in crystallinity index of cellulose [22,32]. Thus, the intensity of impurities peeks is enhanced for the regenerated cellulose-based films. It can also be concluded that both the Film-W and Film-E display similar curves, indicating that the difference of crystalline structure regenerated in different coagulation baths is not obvious in XRD results.

The FTIR spectra of CP, WCCs, and films regenerated in ethanol (Film-E) and deionized water (Film-W) are illustrated in Figure 4b. The WCCs and CP show similar FTIR spectra, but there are minor differences. The WCCs show obvious broad peaks ranging from 1720 cm^−1^ to 1510 cm^−1^, which is ascribed to the overlapped peak of C=O stretching band of hemicellulose (1740 cm^−1^) and aromatic ring stretching band of lignin (1510 cm^−1^) [18,31,46]. Meanwhile, the WCCs also display another characteristic peak of lignin at 1248 cm^−1^ [18,31,46]. Additionally, Film-W and Film-E also exhibit the above characteristic peaks, suggesting that lignin and hemicellulose are still confined in cellulose-based films after WCCs dissolution and regeneration, which corresponds with UV–Vis results. However, the peek at 1110 cm^−1^, which is obvious in spectra of raw materials CP and WCCs, disappears in C-film, Film-W and Film-E. Moreover, the peek at 899 cm^−1^ is weak in the spectra of raw materials, strengthened in that of C-film, Film-W and Film-E. Meanwhile, CP and WCCs exhibit obvious peek at 3275 cm^−1^ and 3281 cm^−1^, respectively, attributed to the O-H stretching band, which shows a blue shift for C-film (3324 cm^−1^), Film-W (3328 cm^−1^), and Film-E (3340 cm^−1^). Similarly, the peak is located at 2896 cm^−1^ for both CP and WCCs ascribed to the C-H stretching band, but it displays a redshift and reaches 2883 cm^−1^ for C-film, and 2881 cm^−1^ for Film-W and Film-E [18]. These results indicate the changes of hydrogen bonds and cellulose crystalline structure, which is consistent with the results of the XRD results. It is worth noting that there are some differences between C-film and cellulose-based films (Film-W and Film-E), indicating that lignin and hemicellulose in Film-W and Film-E may have an impact on the recrystallization of cellulose. In addition to these differences, the WCCs, Film-W and Film-E show similar curves, suggesting that cellulose is the main component, and AmimCl is the nonderivative solvent for WCCs.

### 3.4. Mechanical Property, Hydrophilicity, and Thermal Degradation

**Figure 5:**
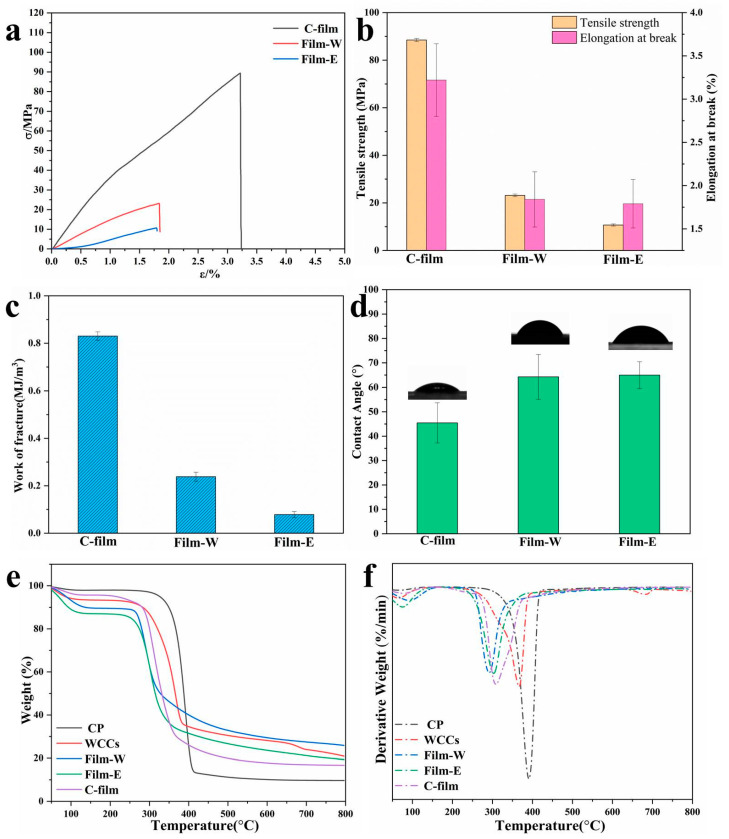
(**a**) Stress–strain profiles, (**b**) tensile strength and elongation at break, and (**c**) work of fracture of C-film, Film-W and Film-E; (**d**) water contact angle of C-film, Film-W and Film-E; (**e**) TG and (**f**) DTG of CP, WCCs, C-film, Film-W and Film-E.

Mechanical property of cellulose-based films decides their application as the packaging materials, and Figure 5a displayed the stress–strain profiles of C-film, Film-W and Film-E. It is worth noting that the degree of polymerization directly decides the tensile strength of polymer material and the DP of CP, and WCCs cellulose is about 530 and 297, respectively, which will have a slight decrease under mild dissolution in AmimCl, as reported before [18], indicating that the cellulose-based films fabricated from WCCs displayed worse mechanical properties than that of cellulose films fabricated form CP. As is shown in Figure 5a, C-film shows higher tensile strength and elongation at break than Film-W and Film-E. However, Film-W and Film-E still possess relatively good mechanical properties. Accordingly, Film-W and Film-E display tensile strength ranging from 10 MPa to 25 MPa (Figure 5a,b). By contrast, Film-W has higher tensile strength (23.16 MPa) and elongation at break (1.84%), compared with those of Film-E (10.68 MPa, 1.79%), suggesting that coagulation bath has a significant influence on the mechanical properties of cellulose-based materials. Furthermore, the work of fracture can be used as an expression of toughness and it is obvious that C-film, Film-W and Film-E show poor toughness (Figure 5c), which is the common phenomenon for cellulose-based films because cellulose macromolecules possess rigid structure and extensive hydrogen bonds [31,32]. In other words, the mechanical property of cellulose-based films fabricated from low-cost WCCs is not good enough, although Film-W has a comparably good mechanical property. However, the strain at break, tensile strength, and toughness of cellulose-based films fabricated from the low-cost source can be improved by adding additives, such as plasticizers [47], microcrystalline cellulose [32], aramid nanofibers [33], etc., which will also add new functions to cellulose films. Generally, the petrochemical polyethylene (PE) film widely used in daily life is flexible and shows tensile strengths in the range of 9–12 MPa [22]. Therefore, the cellulose-based films fabricated from WCCs can be a supplement for petrochemical plastics to address global climate change and promote sustainable development.

Natural cellulosic materials are hydrophilic because of extensive hydrogen bonds, which limit their applications in the packaging industry. As displayed in Figure 5d, the water contact angles (WCAs) of cellulose films are approximately 45.5° (C-film), 64.3° (Film-W), and 66.0° (Film-E), respectively, proving that the cellulose-based films possess good wettability. Moreover, their contact angles are time-dependent (Figure S1). However, Film-W and Film-E exhibit the bigger WCA, because hydrophobic lignin or other impurities are contained in the WCCs and confined in cellulose-based films and the surface of cellulose-based films (Film-W and Film-E) is rougher than that of C-film (Table S1 and Figure S2). Thus, the cellulose-based films fabricated from old waste corrugated cartons display relatively good ultraviolet shielding and hydrophobicity, compared with the traditional pure cellulose film, implying their superiority as packaging and wrapping materials.

Thermal stability is also important for the polymers, so the TGA was carried out to analyze the thermal stability of the raw materials (WCCs and CP) and the cellulose-based materials (C-film, Film-W and Film-E), as presented in Figure 5e,f. It is well known that materials lose moisture firstly when heated, and the mass loss below 200 °C is attributed to the remained moisture loss. It is obvious that the high-quality CP exhibits the highest temperature of maximum weight loss rates (T_max_, 395 °C) and onset decomposition temperature (T_onset_, 280 °C). In contrast, the T_onset_ and T_max_ of WCCs are 255 °C and 365 °C, respectively, lower than those of CP, which is attributed to the smaller DP of WCCs. It is worth noting that the TG curve has a second step, and the DTG curve shows a second T_max_ at around 680 °C, ascribed to the impurities in the WCCs. However, it disappears in the cellulose-based film, suggesting that most of the impurities can be washed partially by the coagulation process. As demonstrated, the C-film, Film-W and Film-E start to degrade above 200 °C, which is ascribed to the decomposition of cellulose macromolecular chains [48]. Furthermore, the lower crystallinity degree of cellulose-based film leads to that T_onset_ and T_max_ of C-film, Film-W and Film-E are lower than that of CP and WCCs, implying that the thermal stability of C-film, Film-W and Film-E decreased sharply after dissolution and regeneration, which was also reported before [48,49]. Film-W has a higher T_onset_ than that of Film-E, while they show comparable T_max_, indicating that the thermal stability is influenced by the coagulation bath. However, the cellulose-based films still have good thermal stability.

### 3.5. Morphology of Cellulose-Based Films

**Figure 6:**
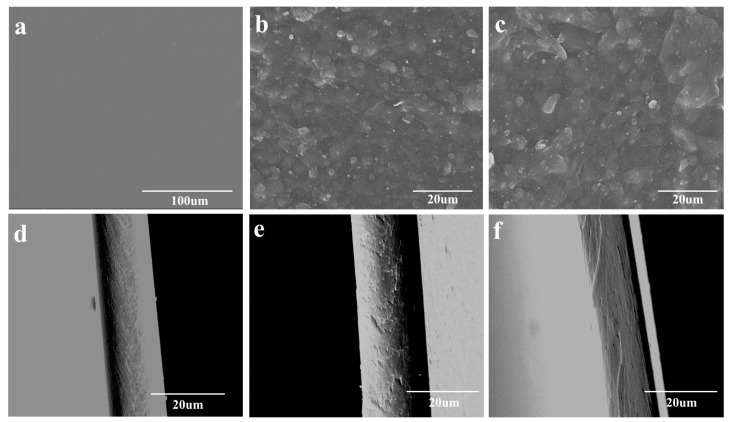
(**a**–**f**) SEM micrographs of C-film, Film-W and Film-E: (**a**–**c**) the surface of C-film, Film-W and Film-E; (**d**–**f**) the cross section of C film, film W, and film E.

The information about the surface and cross-sectional images of cellulose-based films was obtained by scanning electron microscopy, as exhibited in Figure 6. It can be seen that C-film shows the most homogenous and dense microstructure from the surface to the inner (Figure 6a,d). However, both Film-W and Film-E demonstrate a relatively homogenous microstructure and rough surface, because lignin and retained impurities are contained in the films (Figure 6b,c,e,f). Ra, the arithmetical mean roughness can quantifiably describe this difference (Table S1). Moreover, the surface of Film-W (Figure 6b) is smoother than that of Film-E (Figure 6c). Additionally, both Film-W and Film-E display dense inner textures, but Film-W (Figure 6e) shows a more homogeneous cross section than that of Film-E (Figure 6f), implying that the structure of cellulose-based films is obviously influenced by the coagulation process. Therefore, the property and structure of the cellulose materials can be modulated by the coagulated process.

## 4. Conclusions

The semitransparent high-value cellulose-based films were successfully prepared through the AmimCl solvent method by using the inexpensive lignocellulose old waste corrugated cartons as raw materials, where both cations and anions of ILs show synergistic effect in the WCCs dissolution process. It was proved that lignin and other impurities were still confined in the cellulose-based films, and cellulose changed from I to II after the WCCs regeneration process. Compared with traditional pure cellulose film, the cellulose-based films fabricated from WCCs showed better UV-shielding property and hydrophobicity, where the UVA, UVB, and WCA of Film-W and Film-E were 34.68°, 13.9°, 64.3° and 33.42°, 14.14°, and 66.0°, respectively. Meanwhile, Film-W regenerated from deionized water exhibited higher toughness, elongation at break, and tensile strength than those of Film-E regenerated from ethanol. Its tensile strength could reach 23.16 MPa, while the tensile strength of self-sealing bag membrane polyethylene commonly used in the laboratory was 8.95 MPa, lower than that of Film-W. Moreover, the cellulose-based films also possessed good thermal properties, where the T_max_ was at approximately 300 °C. In short, cellulose-based films demonstrate considerable potential for use in the wrapping and packaging industries.

An effective and feasible approach to acquire the valorization of old waste corrugated cartons through fabricating cellulose-based films with high value was developed in this work, where the structure and property of cellulose-based films could be controlled to supplement or even replace the petrochemical plastics. Thus, the low-cost old waste corrugated cartons can be another supplement to the high-quality dissolving pulp, which can comply with sustainable development and tackle global climate change simultaneously.

## Data Availability

The data presented in this study are available on request from the author.

## Supplementary Materials

The following are available online at https://www.mdpi.com/article/10.3390/polym13193359/s1, Figure S1: Time-dependent evaluation of the contact angle of C-film, Film-W and Film-E, Figure S2: Surface profiles of C-film, Film-W and Film-E (sampling length = 5.0 mm), Table S1: The surface roughness of C-film, Film-W and Film-E.
