# Supplementary material for: Cellulose-Based Films with Ultraviolet Shielding Performance Prepared Directly from Waste Corrugated Pulp
Source: Polymers (Basel). 2021 Sep 30;13(19):3359. doi: 10.3390/polym13193359 (PMC8512157; doi:10.3390/polym13193359)
Supplement: Supplementary file 1 [file polymers-13-03359-s001.zip › polymers-1374538-SUP.pdf]

Supplementary Information

Cellulose-based films with ultraviolet shielding performance prepared directly from waste corrugated pulp

Guangmei Xia [1,], Qiwen Zhou[1], Zhen Xu[1], Jinming Zhang[2,*], Xingxiang Ji[1,*], Jun Zhang[2], Haq Nawaz[2], Jie Wang[1] and Jianfeng Peng[1]*

1 State Key Laboratory of Biobased Material and Green Papermaking, Qilu University of Technology, Shandong Academy of Sciences, Jinan, 250353, Shandong, China; biliqwzhou@163.com (Q.Z.); xz521953@qlu.edu.cn(Z.X.); 2711544861@qq.com (J.W.); 1966614429@qq.com (J.P)

2 Beijing National Laboratory for Molecular Sciences, CAS Key Laboratory of Engineering Plastics, Institute of Chemistry, Chinese Academy of Sciences (CAS), Beijing, 100190, China; jzhang@iccas.ac.cn (J. Z.); haqnawaz@bjfu.edu.cn (H.N.)

* Correspondence: gmxia@qlu.edu.cn (G.X.); zhjm@iccas.ac.cn (J.Z.); xxjt78@163.com (X.J.)

C-film

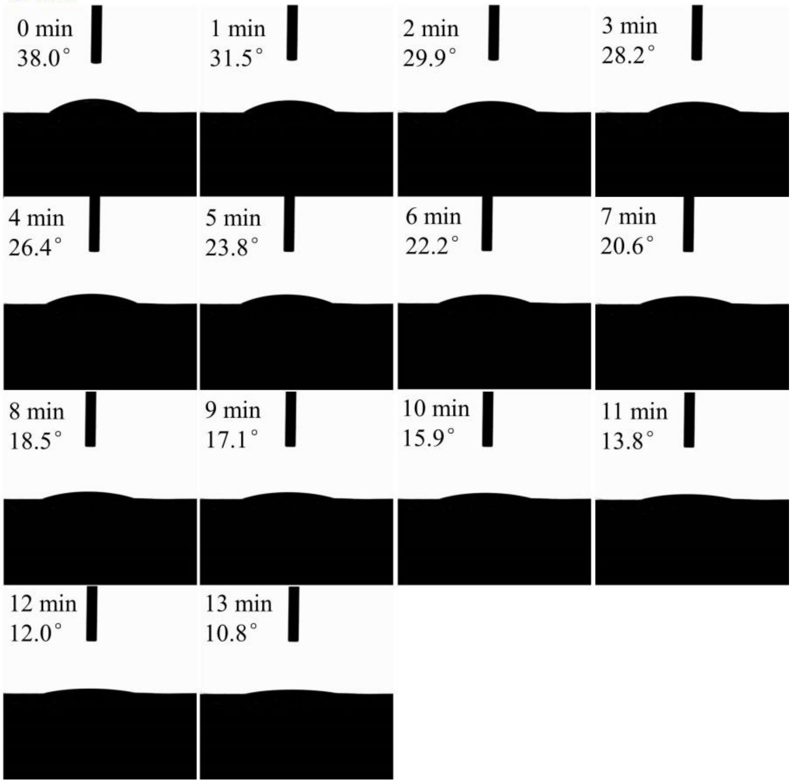

0 min
38.0°
1 min
31.5°
2 min
29.9°
3 min
28.2°
4 min
26.4°
5 min
23.8°
6 min
22.2°
7 min
20.6°
8 min
18.5°
9 min
17.1°
10 min
15.9°
11 min
13.8°
12 min
12.0°
13 min
10.8°

Film-W

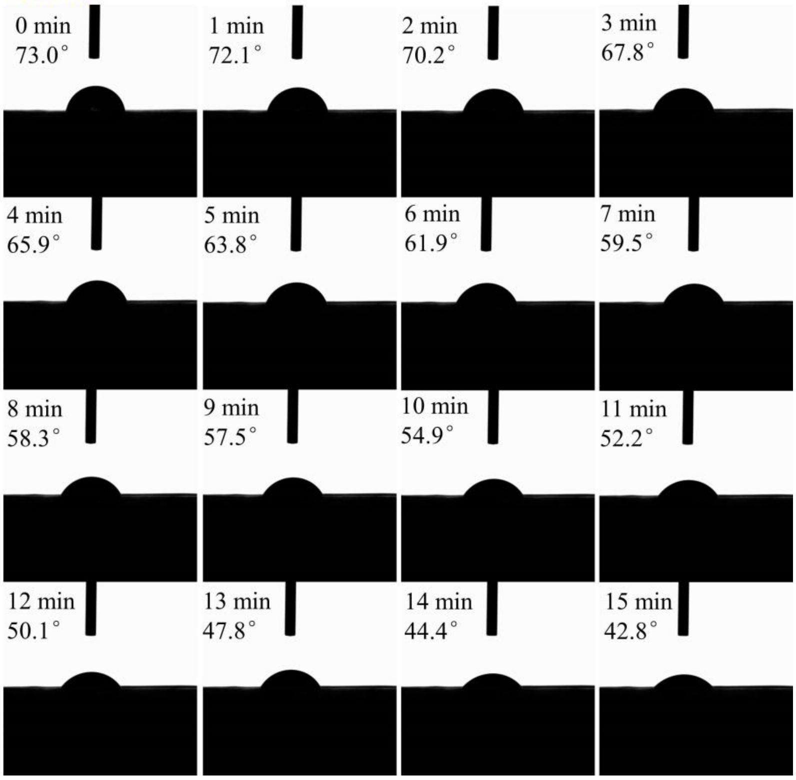

0 min
73.0°
1 min
72.1°
2 min
70.2°
3 min
67.8°
4 min
65.9°
5 min
63.8°
6 min
61.9°
7 min
59.5°
8 min
58.3°
9 min
57.5°
10 min
54.9°
11 min
52.2°
12 min
50.1°
13 min
47.8°
14 min
44.4°
15 min
42.8°

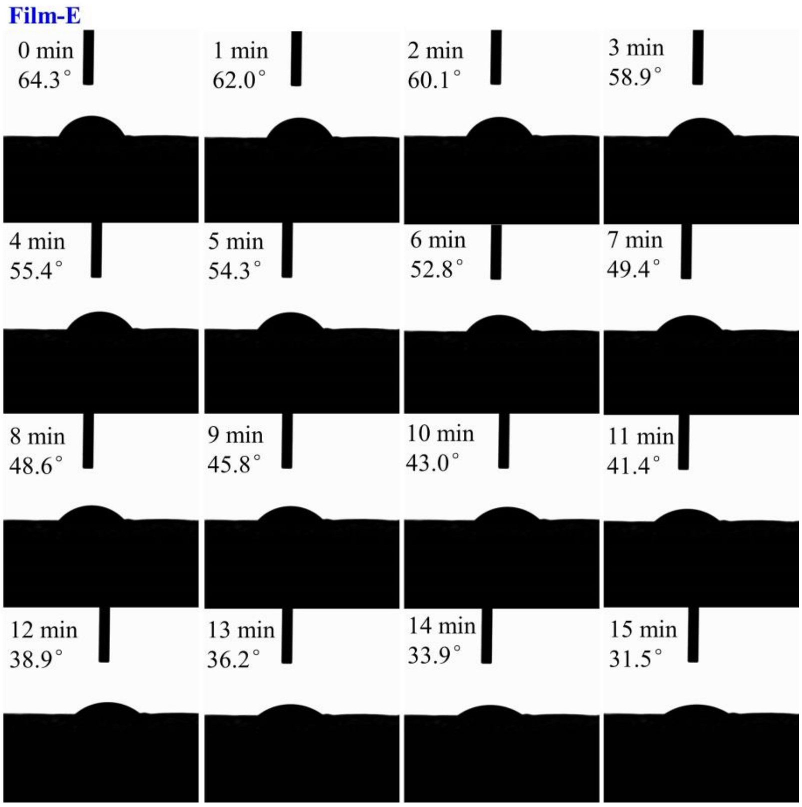

Fig. S1. Time-dependent evaluation of the contact angle of C-film, Film-W and Film-E.

Table S1. The surface roughness of C-film, Film-W and Film-E.

Sample	Surface roughness (Ra, μm)
C-film	0.534 ± 0.077
Film-W	1.874 ± 0.099
Film-E	2.319 ± 0.659

Note: Ra is the arithmetical mean roughness of the film surface recorded with Jitai RT200 roughness measuring instrument.

C-film

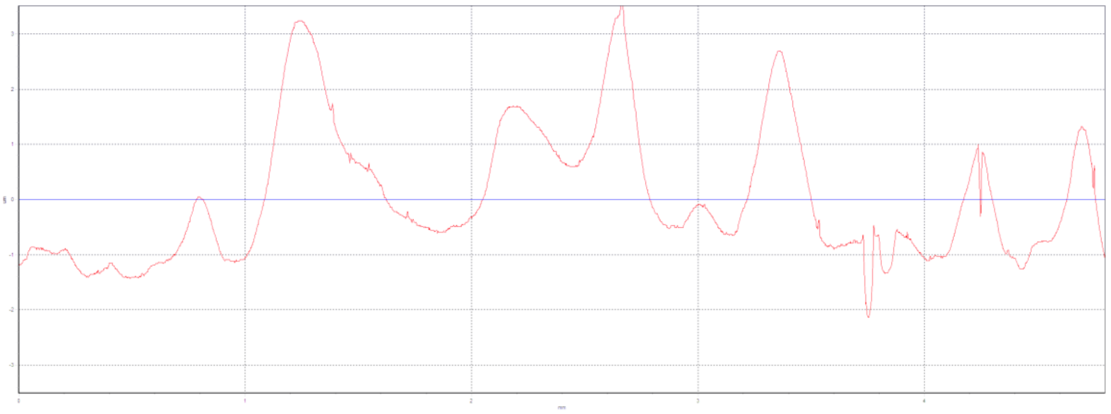

Film-W

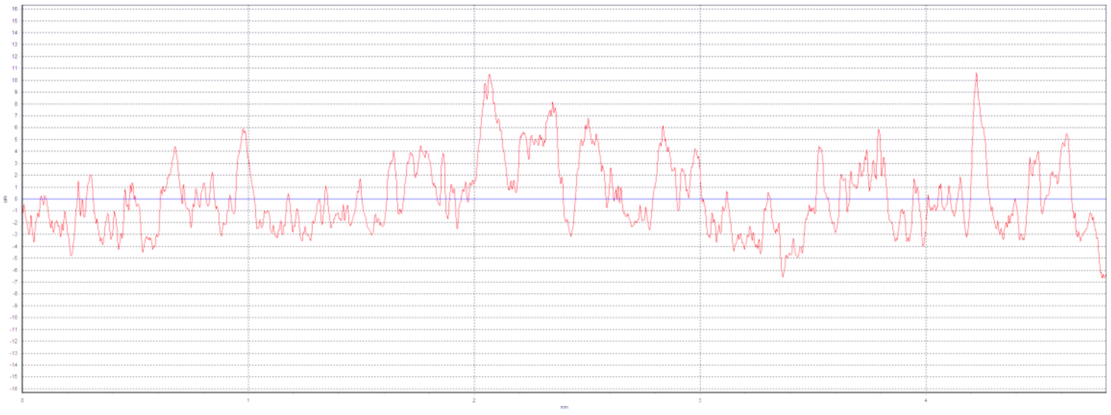

Film-E

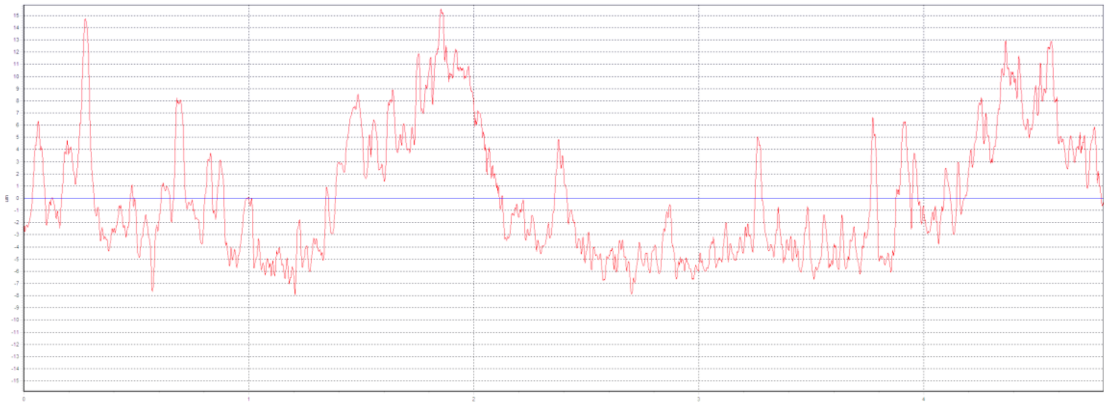

Fig. S2. Surface profiles of C-film, Film-W and Film-E (sampling length=5.0 mm).
